# Close to the same: Similarity influences remembered distance between stimuli

**DOI:** 10.3758/s13423-023-02267-5

**Published:** 2023-03-29

**Authors:** Eileen Pauels, Iris K. Schneider, Norbert Schwarz

**Affiliations:** 1 Technical University Dresden, Dresden, Germany; 2 University of Southern California, Los Angeles, CA USA

**Keywords:** Similarity, Spatial memory, Conceptual metaphor, Spatial processes, Comparison

## Abstract

Six preregistered studies show that the degree of similarity of two objects biases people’s spatial memory of these objects. When objects are high in similarity, people remember having seen them closer together in space than when they are low in similarity. All six studies provide correlational evidence, showing that the more similar participants rated two stimuli, the smaller they remembered the distance between them. This was true for both conceptual and perceptual similarity (Study 3). Furthermore, Studies 2, 4A, and 4B provide evidence of causality by manipulating similarity experimentally. Replicating the correlational findings, highly similar stimuli were remembered as closer together than stimuli low in similarity. This pattern was found across different stimulus categories and similarity dimensions. Overall, these findings show that the similarity of stimuli influences perceivers’ reconstruction of their spatial locations.

**Supplementary Information:**

The online version contains supplementary material available at 10.3758/s13423-023-02267-5.

Similarity judgments are “the very keel and backbone of our thinking” (James, 1890/1995, p. 459). When people talk about similarity, they often reference spatial distance. For all intents and purposes, “The alternatives are very close in value” means the same as “The alternatives are very similar in value”. Such metaphorical expressions are not mere linguistic devices. Instead, such metaphors reflect how people draw on concrete sensory experiences when mentally representing abstract relationships (Barsalou, 2008; Lakoff & Johnson, 1980; Lee & Schwarz, 2014). Demonstrating this, research has shown that distance influences similarity judgments in line with metaphorical expressions: When people see two things close together, they also judge them as being more similar than when they are far apart (Casasanto, 2008; Winter & Matlock, 2013). If distance can make people think about things as more similar, the question arises whether the relationship is bidirectional: Does similarity affect how close or far apart people think two things were? Here, we directly examine this question: Does the conceptual similarity of two objects influence people’s memory of their spatial location? Do they think that two objects were closer to one another in space when the objects were similar rather than dissimilar?

## Similarity and distance

Conceptual metaphor theory posits that people think about abstract concepts in terms of concrete experiences (Lakoff & Johnson, 1980) with consequences for mental representation, judgment, and behavior (for reviews see, Landau, 2017; Landau et al., 2010; Schwarz & Lee, 2019; Thibodeau et al., 2017). One pervasive metaphor is SIMILARITY = PROXIMITY, in which the concrete experience of spatial closeness is used to represent the abstract concept of similarity (Boot & Pecher, 2010; Casasanto, 2008; Winter & Matlock, 2013). For instance, when people say “The two choice options are close” listeners understand that this refers to the similarity of the choice options and not their physical location. Because of this mental association, activating spatial closeness facilitates the processing of similarity information. For instance, when people view two similar squares close together, they are faster to determine that they are indeed similar than when the squares are far apart (Boot & Pecher, 2010). Furthermore, when people see two objects close to each other, they process subsequent sentences about similarity faster than when they first see two objects far apart (Guerra & Knoeferle, 2014).

The spatial distance between things also influences people’s judgments of *how* similar these things are to each other. When people see objects close together, they judge them as more similar compared with when they see the same objects further apart. For example, words set close together are judged to be more similar in meaning than words set far apart (Casasanto, 2008), and stick figures drawn close together are seen as more similar in political views than stick figures drawn far apart (Winter & Matlock, 2013). Importantly, the influence of the SIMILARITY = PROXIMITY metaphor on similarity judgments is limited to conceptual similarity. When people judge tools on their similarity in *use (*conceptual similarity), tools shown close together are judged as more similar than tools far apart. However, when people judge tools on their similarity in *visual appearance*, tools shown close together are judged as less similar than tools shown far apart (Casasanto, 2008).

Does the mental association between distance and similarity mean that similarity also influences distance judgments? Maybe. Studies examining stick figures (Winter & Matlock, 2013) suggest that the SIMILARITY = PROXIMITY metaphor might influence spatial judgments. In one study, participants were presented with descriptions of two students that either emphasized the similarity or dissimilarity of two students. Participants then indicated on a room floor plan where they expected the students would stand during a party. When the descriptions emphasized similarity, participants expected the students to stand closer together than when the descriptions emphasized dissimilarity. Similarly, two cities that were described as similar in their policies and political direction were placed closer together on a map that cities described as dissimilar (Winter & Matlock, 2013). In these studies, the targets’ similarity in personal or political characteristics led to inferences about their likely spatial proximity, a feature about which participants had received no information.

Although compatible with the SIMILARITY = PROXIMITY metaphor, these findings may also be the result of substantive—and empirically correct—assumptions about proximity in daily life. Similar people like one another more (Byrne et al., 1971), and people who like one another stand closer together at a party than people who do not (Baskett et al., 1971). Similarly, cities in the same region are likely to favor similar policies and politics due to shared economic, historical, and cultural influences (Agnew, 1996). In both cases, the observed inferences about the targets’ unknown spatial proximity may therefore reflect reliance on content-specific substantive assumptions about the stimuli rather than, or in addition to, an influence of the SIMILARITY = PROXIMITY metaphor.

### Current studies

We present six studies in which we investigated the relationship between SIMILARITY = PROXIMITY and spatial bias. In all studies, participants were shown two objects that differed in similarity, judged how similar these objects were to one another, and then indicated where they thought the objects had been located when they saw them. Of interest is whether the objects’ conceptual (Studies 1A–4B) or perceptual (Study 3) similarity influences memory for their spatial location, with more similar objects remembered as having been seen closer to one another than less similar objects. We assume that participants draw on the metaphorical association SIMILARITY = PROXIMITY when reconstructing the previously seen locations, resulting in systematic biases in spatial memory.

**Fig. 1:**
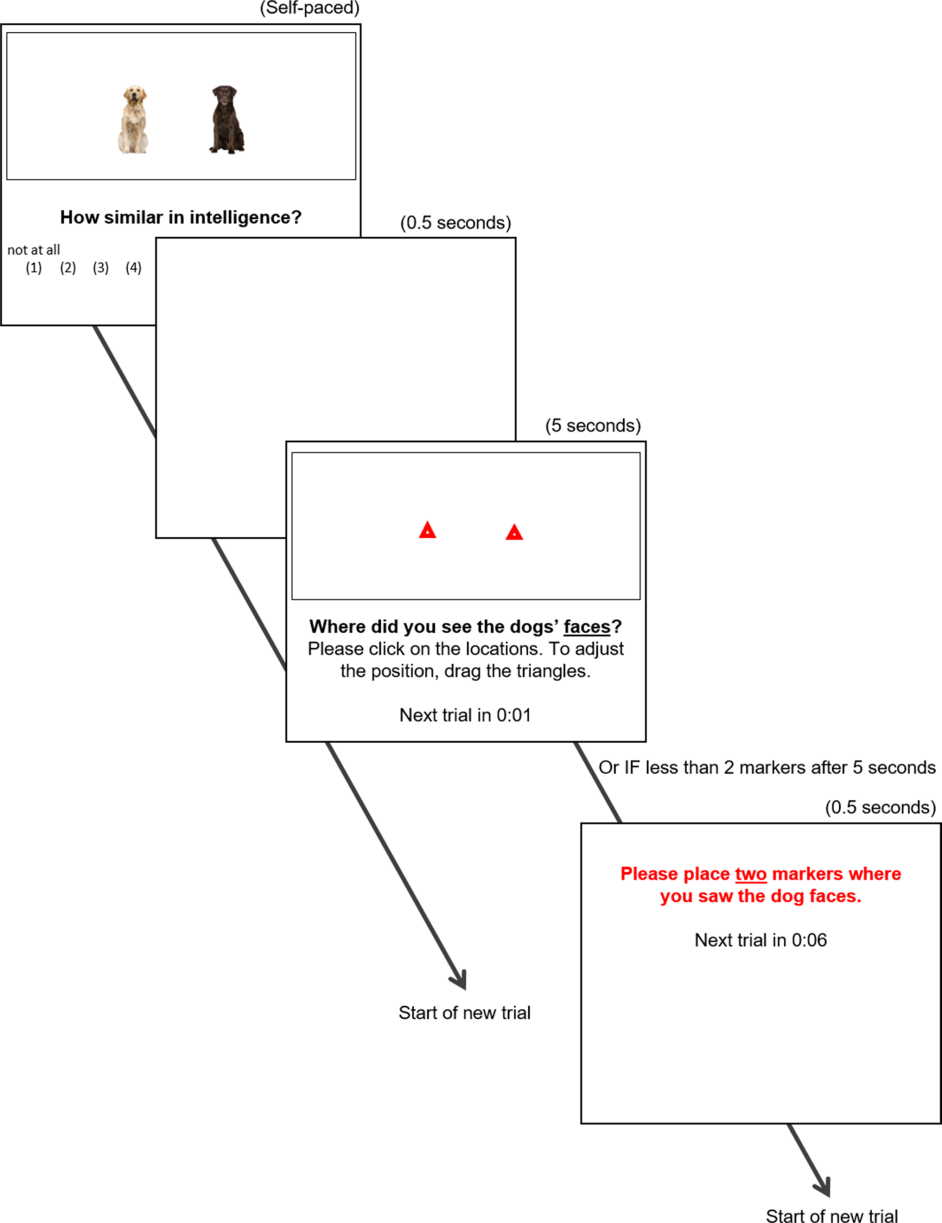
Example of a trial in Study 1A

**Fig. 2:**
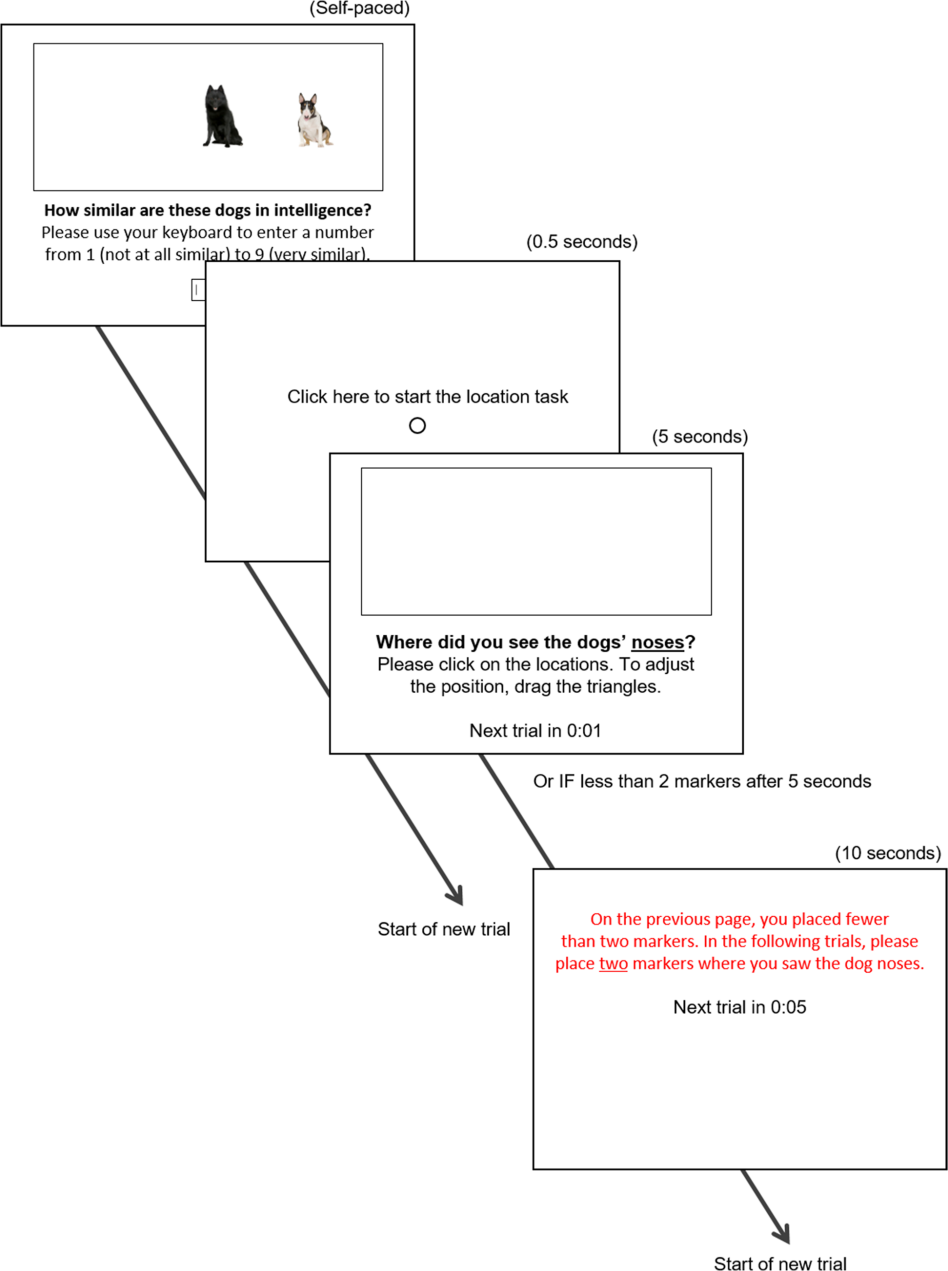
Example of a trial in Study 4A

We tested this hypothesis in correlational (Studies 1A, 1B, and 3) as well as experimental designs (Studies 2, 4A, and 4B), across two different stimulus categories (dogs, food items), three different similarity dimensions (similarity in intelligence, visual appearance, and healthiness), using random stimulus sampling as well as pretested stimuli high or low in similarity. Example stimuli and their presentation format are shown in Fig. 1 and 2. The chosen stimuli, judgment dimensions, and presentation format minimize the likelihood that participants have substantive lay theories about the relationship between the targets’ similarity on the dimension of interest and their spatial proximity in the decontextualized scene shown.

We report all manipulations and measures, as well as all exclusions. All studies were designed on the German survey platform SoSci Survey (Leiner, 2019). All data, material, and preregistrations can be found here: https://osf.io/qye86/.

## Study 1A

In Study 1A, we examined whether the conceptual similarity of stimuli is related to distance memory. We showed participants different pairs of dogs and asked them how similar the dogs were in intelligence. After providing their rating, participants were asked to recall from memory where they had seen each dog on the screen and to mark that location. The distance between the two locations was our main dependent variable. We predicted that the more similar in intelligence the two dogs were perceived, the less distance there would be between the two markers, suggesting a bias in spatial memory in line with the SIMILARITY = PROXIMITY metaphor.

### Method

#### Power

The sample size was set at *N* = 115 to detect a medium bivariate correlation (assumed *r* = .30, one-sided test) with a power of 1 – β = .95. We conducted an a priori power analysis using G*Power 3.1 (Faul et al., 2007).

#### Participants and design

To compensate for data exclusions, we recruited a total of 162 participants through Prolific Academic to participate in a 7-minute survey about judging dogs. We excluded 47 participants based on preregistered exclusion criteria (see Supplementary Materials). In line with the a priori power analysis, the final sample was *N* = 115 (50 men, 65 women; *M*_age_ = 32.70 years, *SD*_*age*_ = 10.96 years; all of UK nationality). Participants received £1.00 for their participation. All participants completed the same procedure, and the variable of interest is the correlation between their similarity judgments and spatial memory.

#### Stimulus material and pair configuration

As stimulus material, we used a set of 71 dog images adapted from Kedia and colleagues (2014) originally retrieved from a commercial online image data base (http://en.fotolia.com/). For each participant, the experimental program randomly drew 52 of those stimuli and matched them into 26 pairs. Thus, each participant saw a different set of stimulus pairs, and each stimulus could appear no more than once per participant. All pairs were presented in an 800-px wide and 300-px high frame (white background, 1-px black border). Each stimulus had a width of 40 px, and we standardized the horizontal distance between the stimuli to 120 px (measured from the inside edges of the stimuli). For each stimulus pair, the two stimuli were presented on the same vertical axis. We varied the position of the stimulus pairs within the frame (Casasanto, 2008; Schneider et al., 2020). That is, each target stimulus pair was randomly presented at one of 26 locations within the frame (see Supplementary Materials for all location configurations).

#### Procedure

At the beginning of the study, we told participants that we were interested in the phenomenon that people need very few cues to judge the character of animals. Participants were told they would see different pairs of dogs and that they would indicate the similarity in intelligence for each pair. They were also told that they would be asked to indicate where they saw the dogs on the screen after each rating. Participants completed four practice trials and 26 target trials.

At the start of each trial, a pair of dogs was presented within the 800-px wide and 300-px high frame (see Fig. 1). Below the frame, participants indicated how similar they thought the dogs were in intelligence (“How similar in intelligence?”; 1 = *not at all* to 10 = *very*). After participants gave their rating, a blank screen appeared for 0.5 seconds to mask the original locations of the stimuli, making it more difficult to remember the exact locations.

Next, participants indicated the locations of the dogs. Because of the semantic association between distance and similarity, we made no verbal reference to “distance.” Instead, we asked participants to indicate where they saw the dogs’ faces (“Where did you see the dogs’ faces?”). To mark the positions of the faces, participants used their mouse to place two red markers within the empty frame. They could adjust the markers’ locations until satisfied with the placement. After 5 seconds, the next trial automatically started. A countdown of the remaining time was presented at the bottom of the page. If a participant did not place two markers within the 5 seconds, an error message that read “Please place two markers where you saw the dog faces” appeared for 10 seconds, after which the next trial would start. Using the *x*- and *y*-coordinates of the markers, we calculated the Euclidian distance between the coordinates using the following formula: $d=\sqrt{{\left({x}_{marker1}-{x}_{marker2}\right)}^2+{\left({y}_{marker1}-{y}_{marker2}\right)}^2}$. This was our main dependent variable After completing 26 trials, participants were asked what they thought the purpose of the study was and answered the exclusion items. Finally, participants had the opportunity to comment on the study and indicated their age and gender before being thanked, debriefed, and compensated.

### Results and discussion

#### Preregistered analyses

We performed multilevel analyses using the nlme package (Pinheiro et al., 2022) in R (R Core Team, 2022) to examine our main hypothesis. We regressed the distance between the stimulus markers on similarity ratings (group centered). As we had no a priori hypothesis assumptions about the model specifications, we compared different model specifications and chose the most parsimonious model with the best model fit (see specifications and Table S2 in the Supplementary Materials). As predicted, the similarity ratings were negatively related to spatial distance, *b* ± *SE* = −1.78 px ± 0.30 px, *t*(2839) = −5.91, *p* < .001.

#### Discussion

Participants remembered dogs as closer together in space the more similar they thought the dogs were in intelligence (see Table 1). On average, the remembered spatial distance shrank by 1.78 pixels for each scale-point increase in judged similarity.

**Table 1:** Overview of all study results

	Intercept	Similarity Rating		Similarity Condition (low vs. high)		Judgment Type (perceptual vs. conceptual)		Similarity Condition* Judgment Type	
Study	*b* (*SE*)	*b* (*SE*)	β (*SE*)	*b* (*SE*)	β (*SE*)	*b* (*SE*)	β (*SE*)		
Study 1A (*N* = 115)	145.77** (1.23)	−1.78** (0.30)	−0.16** (0.03)						
Study 1B (*N* = 119)	142.30** (1.63)	−0.31* (0.14)	−0.04* (0.02)						
Study 2 (*N* = 354)	147.98** (2.00)	−0.78** (0.13)	−0.07** (0.01)	−10.36** (2.58)	−0.40** (010)				
Study 3 (*N* = 328)	142.44** (0.90)	−1.98** (0.17)	−0.19** (0.02)			3.62* (1.79)	0.13** (0.06)	−0.51 (0.33)	−0.05 (0.03)
Study 4A (*N* = 350)	149.22** (2.00)	−1.26** (0.19)	−0.08** (0.01)	−11.67** (2.42)	−0.35** (0.07)				
Study 4B (*N* = 379)	155.86** (1.24)	−0.29** (0.10)	−0.03** (0.01)	−2.49* (0.68)	−0.07** (0.02)				

In all studies we used spatial distance, measured as Euclidean distance (in pixels) between two markers, as the criterion. Study 1A, 1B, and 3 followed a correlational design, while in Study 2, 4A, and 4B, high and low similarity were manipulated. We present unstandardized (*b*) as well as standardized (β) regression coefficients

**p ≤ .001. *p ≤ .05

## Study 1B

In Study 1B, we aimed to replicate Study 1A using a different set of stimuli—namely, food items. Using different stimuli is important to rule out that the relationship between perceived similarity and distance memory is dependent on the kind of stimuli used (Judd et al., 2012; Wells & Windschitl, 1999). The study design was the same as in Study 1A, except for the type of stimuli and the dimension of the similarity judgment. Specifically, we showed participants different pairs of food items and asked them how similar these items were in healthiness. After providing their rating, participants were asked where they had seen each food item on the screen. The distance between the two markers was our main dependent variable. We predicted that the more similar in healthiness the two items were perceived, the less distance there would be between the two markers.

### Method

#### Power

Following the same a priori power analysis as in Study 1A, we aimed for the same sample size as in Study 1A, *N* = 115.

#### Participants and design

To compensate for expected exclusions, we recruited 145 participants through Prolific Academic (prolific.co) for a 7-minute survey about food healthiness. We excluded 26 participants based on the preregistered exclusion criteria (see Table S1 in the Supplementary Materials for details). The final sample was *N* = 119 (38 men, 81 women; *M*_age_ = 36.44 years, *SD*_*age*_ = 12.36 years; all of UK nationality). Participants received £1.20 for their participation. All participants completed the same procedure, and the variable of interest is the correlation between their similarity judgments and spatial memory.

#### Stimulus material and pair configuration

As stimulus material, we used 72 pictures from The Restrain Food Database (Randle et al., 2022). As in Study 1A, for each participant, the experimental program randomly drew 52 stimuli from the stimulus pool and matched them into 26 pairs. The stimulus configurations were equal to Study 1A, such that each random pair was presented in one of 26 possible positions on the screen, and the horizontal distance between the stimuli was 120 px for each stimulus pair (see Fig. S1 in the Supplementary Materials).

#### Procedure

The procedure followed Study 1A, with a few small changes. At the beginning of the study, participants learned that we were interested in the phenomenon that people need very few cues to judge the characteristics of food. To increase variance in the similarity judgments, we added additional information on what constitutes healthy food. We reminded participants of the several nutrients humans need, such as vitamins, minerals, or proteins. We explained that while no one food item can offer all the necessary nutrients, some foods have more nutritious value than others. We also noted that some foods contain nutrients that can be harmful in large amounts, such as sugar, unsaturated fats, and salt.

Participants completed the 4 practice and 26 target trials. In each trial, participants indicated for each pair how similar the food items were in healthiness (“How similar in healthiness?”; 1 = *not at all* to 10 = *very*). On the next screen, they marked where they had seen the food items. To reduce error variance, we instructed participants to mark the location of the center of the food items. After the target trials, participants completed the exclusion criteria and demographical items and had the opportunity to comment on the study. Then, participants were thanked for their participation and forwarded to receive their compensation via Prolific.

### Results and discussion

#### Preregistered analyses

As in Study 1A, we performed multilevel analyses using the nlme package (Pinheiro et al., 2022) in R (R Core Team, 2022), regressing the distance between the stimulus markers on similarity ratings (group centered). In line with the results of the model comparisons conducted for Study 1A (see Supplementary Materials), we ran a random-slopes random-intercept model. As predicted, and replicating Study 1A, the similarity ratings were negatively related to spatial distance, *b* ± *SE* = −0.30 px ± 0.14 px, *t*(2954) = −2.18, *p* = .029.

#### Discussion

The more similar participants judged the food items in healthiness, the closer together they remembered them (see Table 1). On average, the remembered spatial distance shrank by 0.30 pixels for each scale-point increase in judged similarity.

## Study 2

Studies 1A and 1B provided correlational evidence for a relationship between perceived similarity and spatial memory: The more similar two stimuli were perceived to be, the closer to one another they were remembered. To directly test the causal influence of similarity on spatial memory, we experimentally varied stimulus similarity in Study 2. To do so, we presented participants with stimulus pairs that were either high or low in similarity. In line with a SIMILARITY = CLOSE bias in spatial memory, we predicted that high-similarity pairs would be remembered as being closer together than low-similarity pairs. As a manipulation check, we also asked participants to rate each pair’s similarity in intelligence. This rating also allowed us to again examine the correlational relationship between perceived similarity and remembered distance.

### Method

#### Participants and design

To compensate for preregistered exclusions, we recruited a total of 535 participants for a 7-minute survey on dog intelligence on Amazon MTurk via Cloudresearch (cloudresearch.com). One hundred and eighty-one participants were excluded in line with the preregistered exclusion criteria, the large majority because they had two or more missing trials in one of the two similarity conditions (see Table S1 in the Supplementary Materials). Our final sample size was *N* = 354 (209 men, 143 women, two nonbinary people; *M*_age_ = 37.86 years, *SD*_*age*_ = 11.46 years; all of USA residency). Participants received $1.20 for their participation. The study followed a one-factor design with similarity (high vs. low) manipulated within participants and remembered distance as the main dependent variable.

#### Stimulus material

Before the main study, we pretested 69 pairings of the dog pictures used in Study 1 (see Supplementary Materials for details). We chose the 13 pairs with the highest (*M* = 7.04, *SD* = 2.05) and 13 pairs with the lowest (*M* = 4.87, *SD* = 2.37) similarity ratings for the main study. According to a paired-samples t-test, the mean difference between the selected high-similarity pairs and the selected low-similarity pairs was significant, *t*(77) = −12.12, *p* < .001. Furthermore, as indicated by a two-samples Wilcoxon test, the mean objective distances (as measured from dog nose to dog nose) did not significantly differ for high (*M* = 160.77 px, *SD* = 5.66 px) and low-similarity pairs (*M* = 159.96 px, *SD* = 4.18 px, *W* = 69, *p* = .448).

#### Procedure

The study followed the general procedure of Study 1A with a few small changes. As in Study 1B, we added additional information to the study’s introduction. We reminded participants of dogs’ intelligent behaviors, such as learning tricks, understanding words, remembering the location of desired items such as treats, and deceiving other dogs or even humans to get what they want. We noted that although all dogs are capable of some of these behaviors, intelligence varies drastically across dogs. After this, participants started the trials. Half of the trials showed a high-similarity pair, while the other half showed a low-similarity pair. As in the previous studies, participants indicated for each pair how similar the dogs were in intelligence. On the next screen, they marked where they had seen the dogs. We instructed participants to indicate the location of the dogs’ noses (“Where did you see the dogs’ noses?”), which was more specific than the instruction in Study 1A, where we asked for the locations of the dog’s faces.

### Results and discussion

#### Preregistered analyses

Our manipulation of conceptual similarity was successful. Pairs in the high-similarity condition were rated as significantly more similar in intelligence (*M* = 7.52, *SD* = 1.2) than pairs in the low-similarity condition (*M* = 5.25, *SD* = 1.76), *t*(353) = 29.03, *p* < .001, *d*_z_ = 1.52, 95% CI [0.58, 2.45]. To test the predicted causal effect of conceptual similarity on remembered spatial distance, we regressed distance on the similarity condition using multilevel linear regression.^1^ The similarity condition was entered as a dummy-coded fixed factor (low similarity = 0, high similarity = 1), and the distance between stimulus markers (in px) was entered as the criterion. Participants and stimulus pairs were entered as random factors. We estimated the multi-level model in R (R Core Team, 2022) using the packages lme4 (Bates et al., 2015) and lmerTest (Kuznetsova et al., 2017). As predicted, high-similarity pairs (*M* = 137.64 px, *SD* = 17.26 px) were remembered as having been closer to each other than low-similarity pairs (*M* = 148.03 px, *SD* = 16.25 px, *b* ± *SE* = −10.36 px ± 2.58 px), *t*(24.00) = −4.01, *p* < . 001. On average, participants estimated the distance between high-similarity stimuli to be about 10 pixels smaller than the distance between low-similarity stimuli.

Replicating Studies 1A and 1B, we ran a second multilevel model with participants’ individual similarity ratings as a fixed factor and the distance between stimulus markers as the criterion. Again, similarity ratings predicted remembered distance: the more similar a stimulus pair was perceived, the closer in space participants remembered them (*b* ± *SE* = −0.78 px ± 0.13 px), *t*(8575.08) = −6,00, *p* < . 001. Thus, on average, the remembered spatial distance shrank by 0.78 pixels for each scale point the similarity judgment increased.

#### Exploratory analyses: Distance accuracy

We explored whether the conceptual similarity of stimuli also influenced the accuracy of the remembered distance. To do so, we computed an accuracy measure by subtracting the objective distance between the dog noses from the remembered distance. Negative values on this measure indicate an underestimation of distance (in pixels), positive values indicate an overestimation, and zero indicates perfect distance accuracy.

Participants generally *underestimated* the objective distance between the stimuli, but the magnitude differed by similarity condition. Participants underestimated the objective distance between high-similarity pairs by 23.14 pixels on average (*SD* = 17.24 px), *t*(353) = −25.25, *p* < .001, Cohen’s *d* = −1.34, 95% CI [−1.57, −1.11]. They also underestimated the distance between low-similarity pairs, albeit to a smaller extent (i.e., by 11.95 pixels on average; *SD* = 16.24 px), *t*(353) = −13.85, *p* < .001, Cohen’s *d* = −0.74, 95% CI [−0.95, −0.52]. According to the results of a multilevel linear regression of similarity condition on the accuracy measure, the underestimation for high-similarity pairs was significantly higher than for low-similarity pairs, *b* ± *SE* = −11.17 px ± 1.66 px, *t*(−6.75) = 24.01, *p* < .001.

#### Discussion

Study 2 showed that the influence of similarity is causal: Stimuli high in conceptual similarity were remembered as closer in space than stimuli low in conceptual similarity. This memory error was obtained a mere 5.5 seconds or less after the stimuli disappeared from the screen. Study 2 also replicated the correlation between perceived similarity and remembered spatial distance observed in Studies 1A and 1B. Finally, participants underestimated the objective distance in all conditions but the underestimation was twice as large for high-similarity trials.

## Study 3

The preceding three studies show that people’s memory for spatial locations is biased in the direction of the SIMILARITY = PROXIMITY metaphor: The more similar participants thought two dogs were in intelligence, the closer in space they remembered them. The underlying similarity in these studies was conceptual, pertaining to the intelligence of the dogs and the healthiness of the food. In Study 3, we extend our investigation to perceptual similarity, pertaining to visual appearance. Previous work on the effect of the SIMILARITY = PROXIMITY metaphor on similarity judgments (i.e., how distance influences similarity judgments) showed that spatial proximity differentially influences judgments of conceptual versus perceptual similarity, with closeness increasing conceptual similarity but decreasing perceptual similarity judgments (Casasanto, 2008). This asymmetry presumably reflects that the visual properties of stimuli are more easily compared when the stimuli are close together, which facilitates the discovery of differences and hence reduces perceived similarity (Casasanto, 2008). In contrast, the stimuli’s visible properties offer little useful information for conceptual similarity judgments, leading people to rely on heuristics to assess conceptual similarity, including the SIMILARITY = PROXIMITY metaphor (Casasanto, 2008; Schneider & Mattes, 2021).

Whereas this rationale predicts an asymmetrical impact of spatial distance on conceptual versus perceptual similarity judgments, its implications are less clear for the reverse influence of conceptual and perceptual similarity on remembered distance. On the one hand, closer attention to the stimuli’s perceptual features may be associated with a better encoding of the objective distance between them. However, encoding that distance is not necessary for assessing the dog’s perceptual similarity. On the other hand, dogs that are similar in appearance are also often similar in size. Hence, judged similarity of appearance may also serve as a substantively relevant input into the reconstruction of spatial distance between the dogs, similar to our earlier discussion of inferences about the distance between similar people or cities. For that reason, we consider judgments of conceptual similarity a more diagnostic manipulation for testing the role of the SIMILARITY = PROXIMITY metaphor in spatial memory. Study 3 addresses these issues by testing whether conceptual and perceptual similarity differ in their influence on remembered spatial distance.

### Method

#### Power

We conducted an a priori power analysis using G*Power 3.1 (Faul et al., 2007), and sample size was set at *N* = 328 to detect a difference between two independent means (i.e., conceptual vs. perceptual judgment condition) with an effect size of *d* = 0.4 and with a power of 1 − *β* = .95 (two-tailed test, *α* = .05, allocation ratio *N*2/*N*1 = 1).

#### Participants and design

To compensate for exclusions, we recruited 385 participants through Prolific Academic (prolific.co) to participate in an 8-minute survey on judging dogs. We excluded 57 participants based on our preregistered exclusion criteria (see Supplementary Materials), resulting in a final sample size of *N* = 328 (113 men, 212 women, two other, one did not want to disclose their gender; *M*_age_ = 34.36 years, *SD*_*age*_ = 12.73 years; all of UK residency), with 155 participants in the perceptual and 173 participants in the conceptual condition. Participants received £1.20 for their participation. The study followed a one-factor design with judgment type (perceptual vs. conceptual) manipulated between participants and remembered distance as the main dependent variable.

#### Procedure

Following the procedures of Study 1A, participants in the conceptual condition saw 26 dog pairs and rated how similar the members of each pair were in intelligence (1 = *not at all* to 10 = *very*). Participants in the perceptual condition followed the same study procedure but were asked to rate how similar the members of each pair were in physical appearance (1 = *not at all* to 10 = *very*). Remembered distance was assessed as in previous studies. As in Study 2, we asked all participants to mark the location of the dogs’ noses.

### Results and discussion

#### Preregistered analyses

We performed multilevel modeling in R (R Core Team, 2022) using the nlme package (Pinheiro et al., 2022; model specifications in the Supplementary Materials). First, replicating Studies 1 and 2, conceptual similarity was negatively related to remembered distance, *b* ± *SE* = −2.27 px ± 0.24 px, *t*(4263) = −9.32, *p* < .001. The more similar in intelligence the dogs were rated to be, the closer to one another participants remembered them. We found the same pattern for the perceptual similarity condition: The more participants judged the dogs as similar in physical appearance, the closer to one another they remembered them, *b* ± *SE* = −1.71 px ± 0.22 px, *t*(3826) = −7.61, *p* < .001. Regressing distance on similarity rating, judgment type, and their interaction, showed a main effect of similarity as well as of judgment type. The main effect of similarity showed that the more similar two dogs were, the closer they were remembered, *b* ± *SE* = −1.98 px ± 0.17 px, *t*(8089) = −12.00, *p* < .001, replicating our previous findings. Thus, on average, the remembered spatial distance shrank by 1.98 pixels for each scale-point increase in judged similarity, independent of whether the similarity was conceptual or perceptual, *b* ± *SE* = −0.51 px ± 0.33 px, *t*(8089) = −1.55, *p* = .122, for the interaction.

The main effect of judgment type on remembered distance showed that participants who had to judge the similarity of the dogs’ appearance (perceptual similarity) remembered them as having been shown closer together (*M* = 140.62 px, *SD* = 17.26 px) than participants who had to judge the similarity of the dogs’ intelligence (conceptual similarity; *M* = 144.25 px, *SD* = 15.30 px), *b* ± *SE* =3.62 px ± 1.79 px, *t*(326) = 2.02, *p* = .044.

#### Exploratory analyses: Distance accuracy

As in Study 2, the more similar participants perceived two stimuli to be, the more they underestimated the distance between them, *b* ± *SE* = −1.71 px ± 0.17 px, *t*(7768) = −10.18, *p* < .001. For every unit the similarity rating increased, the remembered distance was underestimated by 1.7 additional pixels.

#### Discussion

In sum, conceptual and perceptual similarity had parallel effects on memory for spatial distance. A main effect of similarity indicated that participants remembered more similar dogs as closer together in space, independent of whether they had judged the similarity of the dogs’ intelligence (conceptual) or appearance (perceptual). Investigating the reverse direction of influence, Casasanto (2008) reported that high proximity increased judged conceptual similarity but decreased judged perceptual similarity. He suggested that spatial proximity may facilitate the detection of minor differences, resulting in lower judged similarity of appearance. However, closer attention to details of the appearance of two objects does not necessarily ensure a better encoding of the distance between the objects, which would be required for a beneficial effect on spatial memory. Instead, a main effect of task further indicated that participants remembered the dogs as closer in space after judging their appearance rather than their intelligence. This may reflect that similarity of appearance often entails similarity in size, which is not the case for similarity in intelligence. Hence, appearance similarity may have served as a substantive cue to spatial distance in the context of the present task, resulting in a more pronounced bias after judgments of perceptual rather than conceptual similarity. From this perspective, conceptual similarity provides a more informative test of the assumed influence of the SIMILARITY = PROXIMITY metaphor than perceptual similarity, which confounds the metaphorical influence with the presence of substantive cues. Future research may fruitfully address these conjectures.

## Study 4A

In Study 4A, we addressed two potential methodological concerns. First, in Studies 1A to 3, the horizontal rating scale below the images of the dogs (see Fig. 1) may have served as a mnemonic device for the location of the stimuli, which may have reduced the size of the observed memory bias. Second, in Studies 1A to 3, some participants may have moved the mouse cursor to the area of the stimulus before the screen progressed, keeping it there as a location indicator until the next screen appeared. Although both of these complications would work against the effects observed in Studies 1A to 3, we decided to eliminate them in Study 4A. To do so, we replaced the scale with a text entry box in which participants typed their ratings using the keyboard. We also required participants to click on a button to advance the screen after they made their similarity judgment, thus ensuring that the cursor was in the same screen position for all participants when location markers were requested.

### Method

#### Power

This study is a conceptual replication of Study 2, in which we found a mean difference effect of *d* = .62 between similarity conditions with a sample size of *N* = 350. According to the power analysis tool for models with crossed random effects provided by Westfall et al. (2014), a sample size of *N* = 350 gave us 78% power to detect a medium effect of *d* = 0.62 (stimuli-within-condition design, 350 participants, 26 stimuli, and default VPC settings: Residual VPC = 0.3, Participant intercept VPC = 0.2, Stimulus intercept VPC = 0.2, Participant-by-Stimulus VPC = 0.1, Participant slope VPC = 0.1, Stimulus slope VPC = 0.1). According to the power analysis tool G*Power (Faul et al., 2007), this sample size results in a perfect power (100%) to find a mean difference in a paired-samples *t* test (*d*_z_ = 1.05, one-tailed test, *p* = .001).

#### Participants and design

To compensate for our preregistered exclusions, we recruited a total of 695 participants to participate in a 7-minute survey on dog intelligence on Amazon MTurk via Cloudresearch (cloudresearch.com). Three hundred and forty-five participants were excluded in line with the preregistered exclusion criteria (details in Supplementary Materials), and our final sample size was *N* = 350 (219 men, 130 women, one nonbinary person; *M*_age_ = 38.00 years, *SD*_*age*_ = 10.90 years; all of USA residency). Participants received $1.20 for their participation. The study followed a one-factor design with similarity (high vs. low) manipulated within participants and Euclidian distance as the dependent variable.

#### Stimulus material and procedure

Following the procedures of Study 2, participants saw 13 dog pairs high in similarity and 13 pairs low in similarity and rated how similar the members of each pair were in intelligence. For each pair, participants indicated their similarity judgment (“How similar are these dogs in intelligence?”) by entering a number on their keyboard. To prevent participants from typing two-digit ratings, we restricted the range from 1 (*not at all similar*) to 9 (*very similar*). After participants entered the rating, the study automatically progressed. On the next page, participants saw an empty frame and were asked to click on a fixed location on the screen, resetting the mouse cursor location (see Fig. 2). After clicking, the study continued to the spatial memory task, where participants indicated where they saw the dog’s noses, as in Studies 2 and 3. We adjusted the error message for the location task, which participants saw if they provided less than two markers; the message now read: “On the previous page, you placed fewer than two markers. In the following trials, please place two markers where you saw the dogs’ noses.”

#### Calculation of indicated Euclidian distance

As in the previous studies, we recorded the *x*- and *y*-coordinates (in pixels) of the locations that participants indicated as the remembered position of the dogs’ noses. For our main analysis, we calculated the Euclidian distance ($d=\sqrt{{\left({x}_{marker1}-{x}_{marker2}\right)}^2+{\left({y}_{marker1}-{y}_{marker2}\right)}^2}$ ) between the two markers participants set per trial.

### Results and discussion

#### Preregistered analyses

Our manipulation of conceptual similarity was successful. Pairs in the high conceptual similarity condition were rated as significantly more similar in intelligence (*M* = 6.63, *SD* = 1.48) than pairs in the low conceptual similarity condition (*M* = 4.42, *SD* = 1.70), *t*(349) = 28.57, *p* < .001, *d*_z_ = 1.53, 95% CI [0.59, 2.46]. Using the same mixed-models analyses as in Study 2, high-similarity pairs (*M* = 137.55 px, *SD* = 20.98 px) were remembered as closer to each other than low-similarity pairs (*M* = 149.22 px, *SD* = 21.47 px, *b* ± *SE* = −11.67 px ± 2.42), *t*(24.00) = −4.83, *p* < .001. On average, participants estimated the distance between high-similarity stimuli to be about 11 pixels smaller than the distance between low-similarity stimuli. This replicated the causal effect of similarity on distance memory observed in Study 2, suggesting that the methodological concerns raised concerning Study 2 had no substantial impact. Replicating the correlational findings in Studies 1–3, dogs judged as more similar in intelligence were remembered as presented closer together (*b* ± *SE* = −1.26 px ± 0.19 px), *t*(7308.14) = −6.64, *p* < .001. Specifically, remembered distance shrank by 1.26 pixels with each additional scale point on the similarity scale.

#### Exploratory analyses: Distance accuracy

As in Study 2, we explored whether similarity condition influenced the accuracy of remembered distance. We computed the same accuracy measure as in Study 2 by subtracting the objective distance between the dog noses from the remembered distance.

Results fully replicated the exploratory results of Study 2: Participants underestimated the distance between low-similarity pairs, namely 10.75 pixels on average (*SD* = 21.47 px), *t*(349) = −9.36, *p* < .001, Cohen’s *d* = −0.50, 95% CI [−0.71, −0.29]. They also underestimated the distance between stimuli by twice that amount, *M* = −23.23 pixels on average (*SD* = 20.98 px), *t*(349) = −20.71, *p* < .001, Cohen’s *d* = −1.11, 95% CI [−1.33, −0.88]. The underestimation for high-similarity pairs was significantly higher than for low-similarity conditions, *b* ± *SE* = −12.48 px ± 1.64 px, *t*(24.01) = −7.60, *p* < .001.

As in Study 3, we then explored whether participants’ similarity ratings predicted over- versus underestimation. Replicating the exploratory results of Study 3, the more similar participants perceived two dogs to be, the more participants underestimated the distance between them, *b* ± *SE* = −1.32 px ± 0.19 px, *t*(6351.12) = −6.98, *p* < .001. On average, participants underestimated the distance by an additional 1 pixel per unit increase in the similarity rating.

#### Discussion

In sum, Study 4A replicated the causal effect observed in Study 2 with a different scale format to measure similarity, preventing the possible use of the mouse cursor as a mnemonic aid. This study also replicated the correlational findings from Studies 1A–3, based on perceived similarity rather than similarity condition. Furthermore, the more similar pairs were, the larger the underestimation of distance.

## Study 4B

In Study 4B, we used the food stimuli of Study 1B to replicate the causal effect of similarity perceptions on spatial memory using an entry box instead of a scale. The procedure followed Study 4A, but as in Study 1B, we asked participants to rate how similar food items were in healthiness.

### Method

#### Power

Based on the same power analysis as in Study 4A, we aimed for a total sample size of *N* = 350.

#### Participants and design

To compensate for our preregistered exclusions, we recruited a total of 421 participants to participate in a 7-minute survey on food healthiness on Prolific Academic (prolific.co). Forty-one participants were excluded in line with the preregistered exclusion criteria (as in Studies 1–3; details in Supplementary Materials), which resulted in a final sample size of *N* = 379 (137 men, 239 women, two nonbinary people, one person did not want to disclose their gender; *M*_age_ = 41.56 years, *SD*_*age*_ = 14.43 years; all of UK nationality). Participants received £1.20 for their participation. The study followed a one-factor design with similarity (high vs. low) manipulated within participants and Euclidian distance as the dependent variable.

#### Stimulus material and procedure

As stimulus material, we used 29 pictures from The Restrain Food Database (Randle et al., 2022) used in Study 1A. For low-similarity pairs, we matched one relatively unhealthy food item within one relatively healthy food item (e.g., a doughnut and a piece of broccoli). For high-similarity pairs, we matched either two healthy food items (e.g., salad and cucumber) or two unhealthy food items (e.g., a burger and a muffin). Data from a pretest (*N* = 57) suggested that the 13 preselected low-similarity pairs were indeed perceived as less similar in healthiness (*M* = 2.46, *SD* = 0.83) than the 13 preselected high-similarity pairs (*M* = 7.50, *SD* = 0.84), *t*(56) = 28.46, *p* < .001, *d*_z_ = 3.77, 95% CI [2.77, 4.77] (see Supplementary Materials for further details).

The procedure of the main study then followed that of Study 4A. Accordingly, participants completed four practice trials and 26 target trials: In each trial, first indicating how similar the two food items were in healthiness (same item as in Study 1B: “How similar in healthiness?”) by entering a number of their keyboard (same entry format as in Study 4A, see Fig. 2, valid ratings from 1 = *not at all similar *to 9 = *very similar*); Then, participants clicked on a mark in the middle of the screen to redirect their cursor. Finally, participants indicated where they saw the food items by setting two markers in the empty frame.

### Results and discussion

#### Preregistered analyses

A paired-samples *t* test showed that our manipulation was successful. As in the pretest, participants rated the 13 low-similarity pairs as less similar in healthiness (*M* = 2.20, *SD* = 0.76) than the 13 high-similarity pairs (*M* = 7.41, *SD* = 1.06), *t*(378) = 72.17, *p* < .001, *d*_z_ = 3.71, 95% CI [2.71, 4.71]. To test our main hypothesis, we ran the same mixed-model analysis as in Studies 2 and 4A, treating both participants and stimulus pairs as random factors and using similarity condition as the predictor and the Euclidian distance between the markers as the criterion. Results showed a significant main effect of similarity condition, such that participants remembered high-similarity pairs as closer together (*M* = 154.72 px, *SD* = 23.40) than low-similarity pairs (*M* = 157.20 px, *SD* = 24.20 px, *b* ± *SE* = −2.50 px ± 0.68), *t*(23.99) = −3.65, *p* = .001. On average, participants estimated the distance between high-similarity stimuli to be about 2.5 pixels smaller than the distance between low-similarity stimuli. Furthermore, and replicating all previous studies, a second mixed-model analysis with similarity rating (instead of condition) as the predictor revealed that the more similar two stimuli were perceived to be, the closer they were remembered, *b* ± *SE* = −0.29 px ± 0.10, *t*(68.27) = −2.94, *p* = .005.

#### Exploratory analyses: Distance accuracy

We can also look at these results from an accuracy perspective. Remember that all food stimuli were vertically and horizontally centered on a 40-px wide and 40-px high background. Consequently, the distance between the stimulus centers (i.e., the locations the participants needed to recall) was the same for each pair (i.e., 160 px). Thus, to create an accuracy measure for this study, we subtracted 160 pixels from the remembered distance. As for the accuracy measure previously used, negative values for this measure indicate underestimation, positive values indicate overestimation, and zero indicates perfect accuracy.

Participants *underestimated* the distance between high-similarity pairs by 5.28 pixels on average (*SD* = 23.40 px), *t*(378) = −4.40, *p* < .001, Cohen’s *d* = −0.26, 95% CI [−0.32, 0.09]. They also *underestimated* the distance between low-similarity pairs—namely, by 2.80 pixels (*SD* = 24.20 px), *t*(378) = −2.26, *p* = .03, Cohen’s *d* = −0.12, 95% CI [−0.43, −0.02]. The underestimation for high-similarity pairs was significantly higher than for low-similarity pairs, *b* ± *SE* = −2.50 px ± 0.68, *t*(23.99) = −3.65, *p* = .001.

#### Discussion

Taken together, Study 4B replicates all previous findings, yielding further substantiating evidence for a bias in spatial memory that follows the SIMILARITY = CLOSE metaphor. Moreover, these findings also support a causal relationship between the similarity of two stimuli and the remembered distances between them.

## General discussion

Similarity is one of the core judgments people make when encountering the world around them (Markman & Gentner, 2005; Mussweiler, 2003, 2014; Tversky, 1977). When talking about similarity, people often invoke spatial language, in particular references to closeness. For instance, “These two words are similar in meaning” can be expressed as “These two words are close in meaning” without hurting comprehension. We examined whether the metaphoric association between similarity and proximity influences spatial memory. Across six studies, we consistently found that higher perceived similarity is associated with a smaller remembered distance between stimuli, consistent with the SIMILARITY = PROXIMITY metaphor.

First, the more similar participants rated two stimuli, the smaller they remembered the distance between them (Studies 1A–4B). Second, going beyond this correlational observation, experimental manipulations of similarity showed that highly similar stimuli were remembered as closer together than less similar stimuli (Studies 2, 4A, and 4B). Third, these results were obtained for different types of stimuli (dogs and foods). Finally, these findings held for conceptual similarity (similarity dogs’ intelligence) and perceptual similarity (similarity of dogs’ appearance; Study 3), indicating that both types of similarity can serve as inputs into metaphor-based inferences of proximity. Together, these studies show that spatial memory is biased in line with the SIMILARITY = PROXIMITY metaphor: Stimuli that are higher in similarity are remembered as having been seen closer together in space.

Comparisons of the objective distance at which stimuli were shown and participants’ spatial memory further indicated that participants’ reconstructions consistently underestimated distance. This underestimation was more pronounced for highly similar than for less similar stimuli. Importantly, the underestimation remained significant even at very low levels of perceived similarity (e.g., for stimuli that participants rated as a mere 1 on a 10-point scale; see Supplementary Materials). We suspect that this reflects a systematic difference between specific similarity judgments and the broader ecology of similarity. When asked to judge the similarity of dogs’ appearance or intelligence, the relevant universe consists of dogs, and the ratings reflect within-category comparisons. But considering the diversity of objects in the world, even two dissimilar dogs are more similar to one another than most random pairings of other objects would be. From this perspective, the underestimation of distance despite low-similarity ratings may reflect that the spatial reconstruction is not merely driven by the similarity judgment at hand but also influenced by the similarity of the stimuli in the broader ecological context.

Indeed, all stimulus pairs presented in our studies consisted of exemplars of the same category—participants always judged the similarity of two dogs or two food items. We chose this operationalization because comparisons require that stimuli “have some commensurability” (James, 1890/1995, p. 528) along which the similarity judgment can be aligned (Gentner & Markman, 1994). We assume that the observed effects would be less pronounced for cross-category comparisons, an observation that would be consistent with lower similarity judgments across categories.

As is the case for many conceptual metaphors, the relationship between distance and similarity seems to be reciprocal. Earlier work has addressed the influence of distance on perceived similarity (Casassanto, 2008; Winter & Matlock, 2013) and, relatedly, choice (Boot & Pecher, 2010; Schneider et al., 2020) and categorization (Lakens et al., 2011; Schneider & Mattes, 2021). The present work shows that, conversely, similarity influences distance memory. In combination, these lines of research suggest an intricate relationship between judgments of similarity and distance with potential implications for spatial thinking. We suspect, for example, that the similarity of cities influences estimates of their spatial distance, just as their spatial distance influences inferences about their similarity (Winter & Matlock, 2013). Such mutual influences may affect many aspects of daily life. For instance, estimated travel time might be influenced by the similarity of the destination or people living there, and effects of travel time might influence inferred similarity and related interest in visiting or helping intentions and empathy for other nations.

### Strengths and limitations

In designing the materials for these studies, we deliberately chose nonhuman stimuli. For humans, proximity, similarity, and liking are closely associated for reasons other than the general implications of the SIMILARITY = PROXIMITY metaphor. Physical proximity increases the likelihood of interaction, which fosters increased liking among humans (Festinger et al., 1950; Newcomb, 1961). Interaction as well as liking facilitate social influence, which fosters similarity in attitudes, hobbies, dress, and other variables (Marsden & Friedkin, 1993; McPherson et al., 2001; Newcomb, 1961). Moreover, people who like one another seek closer proximity (Campbell et al., 1966; Little, 1965; Tesch et al., 1973). If human stimuli were used, any of these regularities could serve as an inference rule that suggests that similar people are spatially closer to one another than dissimilar people, rendering the results ambiguous with regard to the general SIMILARITY = PROXIMITY metaphor. As discussed in the context of Study 3, this ambiguity also applies to the pronounced effect of judging appearance similarity, which entails similarity in size, a variable that itself serves as a cue for likely spatial distance in the present paradigm.

Our studies were run in online samples, recruited through crowdsourcing services (Amazon Mechanical Turk and Prolific Academic). This approach provides samples that are more diverse (Castille et al., 2019) and more attentive (Hauser & Schwarz, 2016) than participants in undergraduate subject pools. However, crowdsourcing and online administration provide little control over participants’ hardware and the quality of stimulus presentation. To compensate for that, we carefully designed and preregistered exclusion criteria (for details, see the Supplementary Materials). For instance, we excluded participants whose screens were too small to allow them to see a full trial. In combination with the adoption of best practice recommendations (Hauser et al., 2019), our preregistered exclusion criteria ensured adequate data quality. Multilevel modelling provided additional controls for between-participants variance, including variance contributed by different hardware. In general, higher variability in online data works against the observation of robust and systematic effects in randomized experiments, rendering the concern silent for the consistent results of the six preregistered experiments reported here.

### Conclusion

Initial research into the SIMILARITY = PROXIMITY metaphor focused on the influence of spatial distance on inferred conceptual properties of the stimuli. Stimuli presented closer together are perceived as more similar in meaning (Casasanto, 2008) and more likely to be categorized at a higher level of abstraction (Schneider & Mattes, 2021). Their perceived high similarity also elicits more choice difficulty (Schneider et al., 2020). The present findings highlight the reverse direction of influence: conceptual or perceptual similarity systematically influences remembered spatial distance. This finding is also in line with work showing that the degree to which stimuli are seen as coherent, influences perception of their spatial proximity (von Hecker et al., 2016). In sum: Not only does closeness influence perceptions of similarity, but similarity also influences perceptions of and memory for closeness.

### Supplementary information

ESM 1 (DOCX 86 kb)

## Footnotes

1 This analysis differs from the preregistration for reasons detailed in the Supplementary Materials.
