# Supplementary material for: Close to the same: Similarity influences remembered distance between stimuli
Source: Psychon Bull Rev. 2023 Mar 29;30(5):1814–28. doi: 10.3758/s13423-023-02267-5 (PMC10716182; doi:10.3758/s13423-023-02267-5)
Supplement: Supplementary file 1 — (DOCX 86 kb) [file 13423_2023_2267_MOESM1_ESM.docx]

**Supplementary Materials**

**Exclusion criteria in all studies**

1. Completion: Participants who did not complete the full study or completed fewer than 80% of the trials were excluded.
2. Screen size: Because our visual stimuli were 800 px wide, and particpants would need to be able to see the image in full without scrolling, participants' computer screen width needed to be 800px or more. We recorded screen resolution using a feature provided by our survey platform SoSci Survey (Leiner, 2019) and excluded participants with smaller screen widths.
3. Visibility of frames: In each trial, participants needed to see all relevant information – two stimuli within a frame plus the similarity item below – at the same time. We controlled for this requirement using two items. The first item read: "The image above shows what a complete frame looked like. Considering this, were you able to always see the whole frame, including the dogs and the rating scale?" Participants made a binary choice between "yes, I saw all the dogs [food items] and all the black frames" and "no, the problem was: [free text entry]". We excluded all participants who chose the second choice option. The second item read: "Did you need to scroll down to see the framed pictures and the question below at the same time?". Participants made a binary choice between "Yes, I had to scroll down to see all at once" and "No, I saw the pictures and the question all at once". We excluded all participants who chose the second choice option.
4. Stimulus presentation: We excluded participants who were not able to see the stimuli properly. Specifically, the multiple-select exclusion item read: "Were you able to see the dogs [food items] properly?". Participants could choose any of three choice options: "yes", "no, the pictures were too small: [free text entry]", "no, the picture quality was bad: [free text entry]", and "other problems: [free text entry]". We excluded all participants who did not answer with "yes".
5. Use of measuring aids: Lastly, we exclude participants who used tools to measure the distance between the stimulus pairs. Participants answered one free-text-entry item reading: "Did you use any tools (e.g., your fingers, a ruler, etc.) to remember the location of the dogs' faces [food items]? Your answer will not affect your payment." We excluded all participants who indicated tool usage. In Study 2 to 4A, the item referred to "the dogs' noses" instead of faces.

**Table S1**

***Overview of exclusions per study and per pre-registered exclusion criterion***

| Exclusion Criterion | Study 1A |  | Study 1B |  | Study 2 |  | Study 3 |  | Study 4A |  | Study 4B |
| --- | --- | --- | --- | --- | --- | --- | --- | --- | --- | --- | --- |
| Did not correctly complete  at least 80% of all trials  (i.e., set less than 2 markers) | 6 (3.7%) |  | 1 (0.7%) |  | 113 (21.1%) |  | 10 (2.6%) |  | 200 (28.9%) |  | 4 (1.0%) |
| Screen width < 800 px | 28 (17.3%) |  | 3 (2.1%) |  | 3 (0.6%) |  | 6 (1.6%) |  | 0 (0.0%) |  | 4 (1.0%) |
| not able to see the full frame including rating scale  at the same time | 1 (0.6%) |  | 2 (1.4%) |  | 1 (0.2%) |  | 4 (1.0%) |  | 4 (0.6%) |  | 0 (0.0%) |
| Had to scroll to see a full trial | 1 (0.6%) |  | 3 (2.1%) |  | 38 (7.1%) |  | 2 (0.5%) |  | 124 (17.9%) |  | 5 (1.2%) |
| Indicated insufficient  stimulus quality | 3 (1.9%) |  | 4 (2.8%) |  | 4 (0.8%) |  | 4 (1.0%) |  | 4 (0.6%) |  | 14 (3.3%) |
| Used tools to measure the  distance between stimuli | 8 (4.9%) |  | 13 (9.0%) |  | 15 (2.8%) |  | 31 (8.1%) |  | 13 (1.9%) |  | 15 (3.7) |
| Total number of exclusions | 47 (29.0%) |  | 26 (17.9%) |  | 181 (33.8%) |  | 57 (14.8%) |  | 345 (49.8%) |  | 42 |
| Total number of complete datasets | 162 |  | 145 |  | 535 |  | 385 |  | 695 |  | 421 |
| **Sample size after exclusions** | **115** |  | **119** |  | **354** |  | **328** |  | **350** |  | **379** |

*Note.* We administered the exclusion criteria consecutively (top to bottom row). The percentages (in brackets) are based on the number of complete datasets.

**Configuration of stimulus locations in all studies**

In all six studies, stimulus pairs were presented in one of 26 locations within the frame, with each location presented once per participant. Figure S1 shows all possible horizontal configurations. For example, configuration A in Figure S1 showed the left stimulus 60 pixels from the left frame border, and the right stimulus 220 pixels from the left frame border. Each horizontal configuration was presented twice to each participant, once 30 pixels from the bottom frame border, and once 50 pixels from the bottom frame border. Accordingly, for each participant, each of the 26 stimulus pairs was presented at a unique position. The positions were used in random order.

**Figure S1**

***Stimulus pair configurations
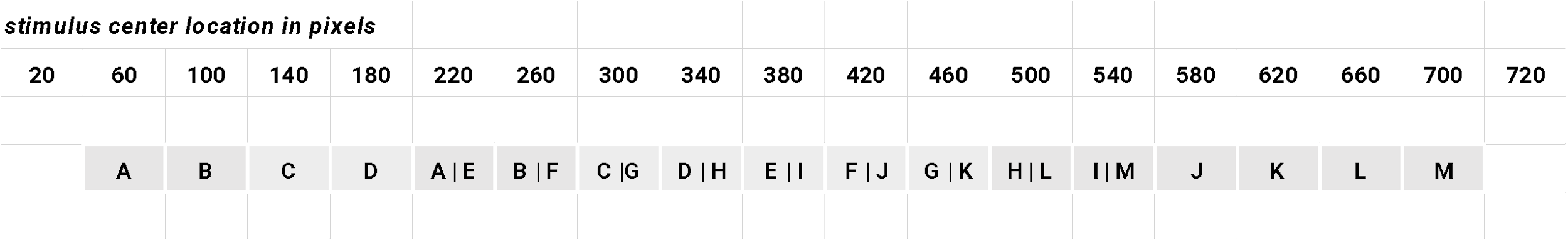
***

*Note*. In all studies, stimulus pairs were presented in a frame with white background and a 1px black border (width: 800px, height: 300px). Each of the 13 letter pairs (e.g., A & A) refers to one of 13 possible horizontal locations of a stimulus pair within the frame. We used each of the horizontal locations twice, once with a 50px distance from the bottom frame border and once with a 30px distance from the bottom frame border.

**Study 1A: Multilevel model specification**

In Study 1, we regressed the distance between the stimulus markers on the participant intercept as well as similarity ratings using a multilevel linear modeling approach. We set participant as level 2 unit, and trial as level 1 unit. We centered perceived similarity at the participant mean. We computed models of different complexity, building them up from the intercept-only model. We compared all models using deviance tests and chose the model with the best fit to our data, i.e., a random-slopes model with perceived similarity as the predictor. Table S2 shows all model specifications, model comparisons, and regression weights.

**Table S2**

***Multilevel modelling Study 1A, regressing remembered distance on perceived similarity***

|  |  | Model 1  Intercept Only | | |  | Model 2  Random Intercept | | |  | Model 3  Random Intercept + Perceived Similarity | | |  | Model 4  Random Intercept/Slopes +  Perceived Similarity | | |
| --- | --- | --- | --- | --- | --- | --- | --- | --- | --- | --- | --- | --- | --- | --- | --- | --- |
| Variable |  | *B* | *SE* | *p* |  | *B* | *SE* | *p* |  | *B* | *SE* | *p* |  | *B* | *SE* | *p* |
| Predictor statistics | | | | | | | | | | | | | | | | |
| Intercept |  | 145.79 | 0.47 | < .001 |  | 145.78 | 1.23 | < .001 |  | 145.77 | 1.23 | < .001 |  | 145.77 | 1.23 | < .001 |
| Perceived Similarity^a^ |  |  |  |  |  |  |  |  |  | -1.61 | 0.22 | < .001 |  | -1.78 | 0.30 | < .001 |
| Model statistics | | | | | | | | | | | | | | | | |
| -2 Log-likelihood |  | 27523.50 |  |  |  | 26965.94 |  | < .001 |  | 26910.24 |  | < .001 |  | 26889.20 |  | <.001 |
| df |  | 2 |  |  |  | 3 |  |  |  | 4 |  |  |  | 6 |  |  |
| AIC |  | 27527.50 |  |  |  | 26971.94 |  |  |  | 26918.24 |  |  |  | 26901.20 |  |  |
| *Note.* Model statistics compare each model to the previous, more parsimonious one.  ^a^ Values centered at the participant mean. | | | | | | | | | | | | |  |  | | |

**Study 2: Pretest**

We pretested 69 pairs of pictures of dogs for their perceived similarity in intelligence. The goal was to identify pairs perceived to be most similar in intelligence and those perceived to be least similar in intelligence. We then used these pairs in Study 3 to manipulate perceived similarity.

***Participants***

We recruited 101 participants on Amazon MTurk via Cloudresearch to participate in a 10-minute survey about dog intelligence. We excluded 2 participants because they used a screen with less than 800px width and another 21 participants because they indicated that they could not see the full-frame, including the rating scale, without scrolling. Thus, our final sample size was *N* = 78 (45 men, 32 women, 1 chose not to disclose their gender; *M*_age_ = 40.76, *SD_age_* = 10.66). Participants received $1.60 for their participation.

***Stimulus material***

As stimulus material, we used 16 of the dog pictures used in Study 1A. We created 69 different pairs from these 16 pictures.

***Procedure***

Participants read the same introductory information as in Study 1A. Then, participants saw all stimulus pairs in random order. As in the previous studies, each stimulus pair was presented in a frame of 750px width and 300px height, with a white background and a 1px black border. All pairs were presented in the center of the frame, with 200px distance between them (i.e., the center of the left stimulus was 300px from the left border, and the center of the right stimulus was 500px, stimulus width = 40px). For each pair, participants indicated how similar they thought the two dogs were in intelligence (1 item: "How similar in intelligence?", scale ranged from 1 (*not at all*) to 10 (*very*)). After completing all sixty-nine trials, we administered our exclusion items (same as in the previous studies, see Supplementary Materials). Lastly, participants were able to comment on the study and indicated their age and gender. Finally, participants were thanked, debriefed, and compensated.

***Results***

We computed the average scores of perceived similarity for each stimulus pair. We then computed a paired samples t-test on perceived similarity between the thirteen lowest scoring and the thirteen highest-scoring pairs. Results showed that participants rated the pairs in the high-similarity subset (*M* = 7.06, *SD* = 0.29) to be more similar than the pairs in the low-similarity subset (*M* = 4.86, *SD* = 0.28, *t*(77) = -12.12, *p* < .001). Thus, we decided to use these two subsets as similarity manipulation in the main study.

**Study 2: Pre-registered analysis**

Originally, we pre-registered to control for the real distance between the stimuli in a repeated-measures ANCOVA with similarity condition (high vs. low, within-subjects) as IV and distance between the stimulus markers as the DV. However, controlling for the real distance in a mean difference test would not have added any meaningful information, as the mean actual distance between high-similarity and low-similarity pairs was held constant for all participants. In other words, as all participants saw the same high-similarity as well as low-similarity pairs, the mean actual distance perfectly correlates with the similarity condition. Note that an unpaired two-samples Wilcoxon test on the true distances per similarity condition showed that on a mean level, high-similarity pairs were equally far apart (*M* = 160.77, *SD* = 5.66) as low-similarity pairs (*M* = 159.96, *SD* = 4.18, *W* = 69, *p* = .448).

In a multilevel linear model like reported as our main analysis, one can control for the actual distance between stimuli on a trial-by-trial basis. To do so, we ran an additional random-intercept-random-slopes model, adding true distance as a covariate. Results showed that true distance between stimuli also predicted remembered distance: the higher the distance between the dogs’ noses, the higher the remembered distance, *b* ± *SE* = 1.02px ± 0.18px, *t(*23.01) = 5.75, *p* < .001. More importantly, however, adding the covariate did not change our main finding that similarity condition predicted remembered distance: That is, independent of the true distance between stimuli, high-similarity pairs were perceived to be closer together than low-similarity pairs, *b* ± *SE* = -11.19px ± 1.70px, *t*(23.01) = -6.59, *p* < .001.

**Study 3:** **Multilevel model specification**

In Study 3, we regressed remembered distance on the participant intercept, perceived similarity, similarity type condition, as well as the interaction between the two latter predictors. We used a multilevel linear modeling approach, using participant as level 2 unit, and trial as level 1 unit. We centered perceived similarity at the participant mean. Also, we dummy-coded similarity type condition, such that 0 corresponds to the perceptual condition, and 1 corresponds to the conceptual condition. We computed models of different complexity, building them up from the intercept-only model. We compared all models using deviance tests and chose the model with the best fit to our data, i.e., a random-slopes model with perceived similarity as the predictor. Table S3 shows all model specifications, model comparisons, and regression weights.

**Table S3**

***Multilevel modeling Study 3, regressing remembered distance on perceived similarity condition, similarity type condition, and their interaction***

|  |  | Model 1  Intercept Only | | |  | Model 2  Random Intercept | | |  | Model 3  Random Intercept + Perceived Similarity | | |  | Model 4  Random Intercept/Slopes +  Perceived Similarity | | |  | Model 5 Random Intercept/Slopes + Perceived Similarity + Similarity type | | | |
| --- | --- | --- | --- | --- | --- | --- | --- | --- | --- | --- | --- | --- | --- | --- | --- | --- | --- | --- | --- | --- | --- |
| Variable |  | *B* | *SE* | *p* |  | *B* | *SE* | *p* |  | *B* | *SE* | *p* |  | *B* | *SE* | *p* |  | *B* | *SE* | *p* | |
| Predictor statistics | | | | | | | | | | | | | | | | | | | | | |
| Intercept |  | 142.58 | 0.30 | <.001 |  | 142.54 | 0.90 | <.001 |  | 142.54 | 0.90 | <.001 |  | 142.54 | 0.90 | <.001 |  | 142.54 | 0.90 | <.001 | |
| Perceived similarity^a^ |  |  |  |  |  |  |  |  |  | -1.90 | 0.14 | <.001 |  | -1.99 | 0.17 | <.001 |  | -1.98 | 0.16 | <.001 | |
| Similarity type^b^ |  |  |  |  |  |  |  |  |  |  |  |  |  |  |  |  |  | 3.62 | 1.79 | .044 | |
| Perceived similarity * similarity type |  |  |  |  |  |  |  |  |  |  |  |  |  |  |  |  |  | -0.51 | 0.33 | .122 |  |
| Model statistics | | | | | | | | | | | | | | | | | | | | |  |
| -2 Log-likelihood |  | 79898.48 |  |  |  | 77544.58 |  | <.001 |  | 77361.28 |  | <.001 |  | 77344.30 |  | <.001 |  | 77337.58 |  | .035 |  |
| df |  | 2 |  |  |  | 3 |  |  |  | 4 |  |  |  | 6 |  |  |  | 8 |  |  |  |
| AIC |  | 79902.48 |  |  |  | 77550.57 |  |  |  | 77369.28 |  |  |  | 77356.30 |  |  |  | 77353.59 |  |  |  |
| *Note.* Model statistics compare each model to the previous, more parsimonious model.  ^a^ centered at the participant mean  ^b^ dummy-coded, 0 = perceptual condition, 1 = conceptual condition | | | | | | | | | | | | | | | | | | | | |  |
|  | | | | | | | | | | | | | | | | | | | | |  |

**Study 4A: Accuracy analysis per similarity scale point**

To see whether participants significantly underestimated the distance between stimuli even at low levels of perceived similarity, we ran a one-sided one-sample t-test on the accuracy score per scale point of the similarity ratings. Results showed that participants significantly underestimated the distance between stimuli at any given scale point (all *p*s <. 001). Table S4 shows the results in full.

**Table S4**

| Scale point | *M* (*SD*) |  | *df* |  | *t* |  | *p* |
| --- | --- | --- | --- | --- | --- | --- | --- |
| 1 (not at all similar) | -12.20 |  | 580 |  | -8.16 |  | <.001 |
| 2 | -8.38 |  | 637 |  | -5.45 |  | <.001 |
| 3 | -12.01 |  | 788 |  | -11.05 |  | <.001 |
| 4 | -12.04 |  | 920 |  | -8.75 |  | <.001 |
| 5 | -15.82 |  | 1284 |  | -19.14 |  | <.001 |
| 6 | -17.23 |  | 1220 |  | -19.58 |  | <.001 |
| 7 | -19.24 |  | 1250 |  | -21.65 |  | <.001 |
| 8 | -22.32 |  | 1223 |  | -26.09 |  | <.001 |
| 9 (very similar) | -25.53 |  | 1015 |  | -24.13 |  | <.001 |

*Study 4: Results from one-sample t-tests on accuracy scores per similarity scale point*

**Study 4B: Pretest**

We pretested two sets of pairs of pictures of food items for their perceived similarity in healthiness. The goal was to test prior to the main study (Study 4B) that pairs in one subset would be perceived as more similar in healthiness than pairs in the other subset. We then used these pairs in Study 4B to manipulate perceived similarity.

***Participants***

We recruited 62 participants on Prolific Academic to participate in a 4-minute survey about food healthiness. We excluded 5 participants based on the same exclusion criteria as in the main studies (except for tool usage and the 80% completion criterion, as they were not applicable in this pretest). Thus, our final sample size was *N* = 57 (19 men, 32 women, 1 chose not to disclose their gender; *M*_age_ = 35.93, *SD_age_* = 11.92). Participants received £0.70 for their participation.

***Stimulus material***

As stimulus material, we used 29 of the food pictures used in Study 1B. We created 26 different pairs from these pictures. Most of the pictures were used both in the high-similarity and in the low-similarity condition. An overview of the stimulus material can be found on OSF.

***Procedure***

Participants read the same introductory information as in Study 1B. Then, participants saw all stimulus pairs in random order. As in the previous studies, each stimulus pair was presented in a frame of 750px width and 300px height, with a white background and a 1px black border. All pairs were presented in the center of the frame, with 200px distance between them (i.e., the center of the left stimulus was 300px from the left border, and the center of the right stimulus was 500px, stimulus width = 40px). For each pair, participants indicated how similar they thought the two food items were in healthiness by entering a one-digit number into a text box (1 item: "How similar in healthiness?", response options from 1 (*not at all*) to 10 (*very*), see Study 4A and 4B). After completing all 26 trials, we administered our exclusion items (same as in the previous studies, see Supplementary Materials). Lastly, participants were able to comment on the study and indicated their age and gender. Finally, participants were thanked, debriefed, and compensated.

**Results**

Mean comparison with a paired samples t-test showed that high-similarity pairs were indeed rated to be more similar in healthiness (*M* = 7.50, *SD* = 0.84) than low-similarity pairs (*M* = 2.46, *SD* = 0.83; *t*(56) = 28.46, *p* < .001). Thus, we conclude that our similarity manipulation has worked.

To investigate the results further, we looked at each stimulus pair separately. Descriptively, all of the pairs in the high-similarity subset were perceived as more similar than the pairs in the low-similarity subset. We tested whether the highest-rated low-similarity pair (Pair 13, *M* = 4.42, *SD* = 2.46) was perceived as significantly less similar than the lowest-rated high-similarity pair (Pair 5, *M* = 6.68, *SD* = 2.07). A paired-sampled t-test revealed that the difference in similarity ratings was statistically significant, *t*(56) = 5.90, *p* < .001. We thus concluded that we could use all selected stimulus pairs for the main study (i.e., Study 4B).
